# Homonymous hemianopsia as the leading symptom of a tumor like demyelinating lesion: a case report

**DOI:** 10.1186/1757-1626-2-9366

**Published:** 2009-12-21

**Authors:** Maria Eleptheria Evangelopoulos, Dimitrios Stergios Evangelopoulos, Costas Potagas, Costantinos Sfagos

**Affiliations:** 1 Department of Neurology, University of Athens, Aiginitio University Hospital, Athens, Greece; 2 Department of Orthopaedic, University of Athens, KAT Accidents' Hospital, Athens, Greece

## Abstract

**Introduction:**

Differential diagnosis of a cerebral lesion can prove to be a very challenging task for the treating physician. Many non-neoplastic neurological diseases can mimic brain neoplasms on neuroimaging.

**Case presentation:**

A previously healthy 23-year-old male, presented with blurred vision to the Emergency Department of our Hospital. After initial clinical and serological examination, he was admitted to our clinic for further investigation. Neurological examination showed left homonymous hemianopsia. Brain MRI revealed edema of the right parietal lobe, compressing the posterior region of the right ventricle. Serum viral, immunological and paraneoplasmatic testing were negative. Spectroscopic MRI described the lesions as tumefactive demyelinated plaques. After treating the patient with intravenous corticosteroids, his symptoms rapidly improved and the extensive lesion of the parietal lobe decreased.

**Conclusion:**

In case of young patients with tumor-like lesions, demyelination should always be considered in the differential diagnosis.

## Introduction

Differential diagnosis of a cerebral lesion can prove to be a very challenging task for the treating physician. Many non-neoplastic neurological diseases including multiple sclerosis, stroke, pyogenic abscess, progressive multifocal leukoencephalopathy, acute disseminated encephalomyelitis, toxoplasmosis, tuberculosis, cysticercosis, fungal infections, syphilis, sarcoidosis, Behçet disease, radiation necrosis, venous thrombosis can mimic brain neoplasms on neuroimaging [1,2]. Some of these diseases have a benign character and can be managed without exposing patients to the unnecessary risks of a surgical procedure [3,4]. On the other hand, certain brain tumors can appear de novo, in the absence of prior existing lesions, mimicking other diseases [5,6]. Therefore, an accurate and timely diagnosis is essential in such cases and special techniques are required for proper differential diagnosis.

We present the case of a patient, without a previous history of multiple sclerosis, presenting with a large brain lesion mimicking a brain tumor. We discuss the diagnostic process and techniques in the management of tumefactive Multiple Sclerosis.

## Case presentation

A previously healthy 23-year-old Caucasian male was referred to our Emergency Department. Upon admission, he reported a 3-weeks old history of blurred vision and instability, lasting for 5 days.

On physical examination, patient's vital signs, ECG and general medical condition were normal. He was fully alert and oriented. Neurological examination revealed a left homonymous hemianopsia. Tendon reflexes were equally elicited on both sides for upper and lower limbs and a Babinski sign was detected on the left side. There was not any bowel or bladder dysfunction. No other motor or sensory deficit was found in the Neurological examination.

**Figure 1:**
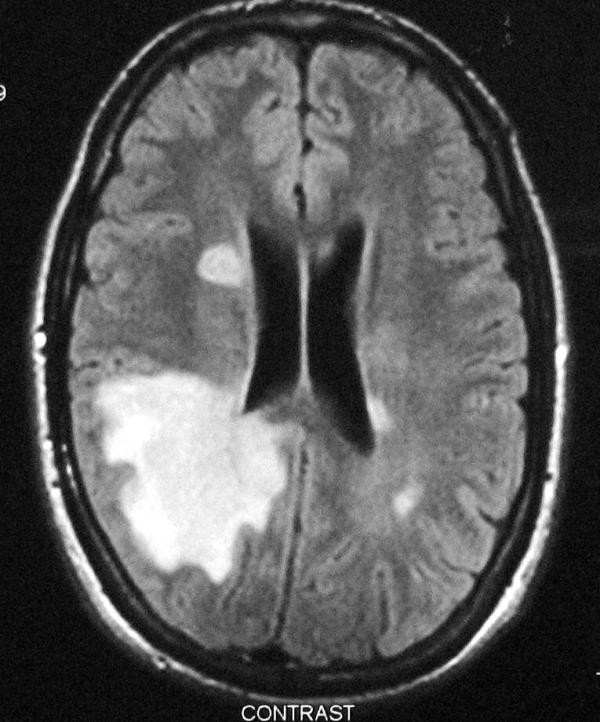
**MRI upon admission: T2W & Flair sequences revealed a lesion extending from the right parietal to the temporal lobe, compressing the posterior aspect of the right ventricle**. No lesion showed gadolinium enhancement.

Brain MRI presented a large lesion at the right parietal lobe extending till the temporal lobe with consequent edema, compressing the posterior aspect of the right ventricle. Few small periventricalar lesions were found in both cerebral hemispheres as well as in the cerebellum. No lesion showed gadolinium enhancement (Figure 1). Staging with thoracic and abdominal CT scans had not revealed any other lesions. Cervical MRI did not detect any spinal cord lesions.

Therefore, intracranial lesions such as tumors (CNS Lymphoma), abscess, sarcoidosis and demyelinating disease should lead the differential diagnosis.

Patient's routine blood tests were within normal range. Mantoux was negative. Serum Sace was normal. Electrophoresis and immunoelectrophoresis were mot indicative of any pathology. CSF examination showed pleiocytosis (92 cells), mostly lymphocytes and few mononuclear cells while IgG Index was increased and oligoclonal bands were positive. Serum viral examination for HIV, HBV, HCV, HSV1, HSV2 and CMV was negative, while VZV IgG were detected at remarkably high titers (VZV IgG: 784.84 (positive: normal values < 60)

**Figure 2:**
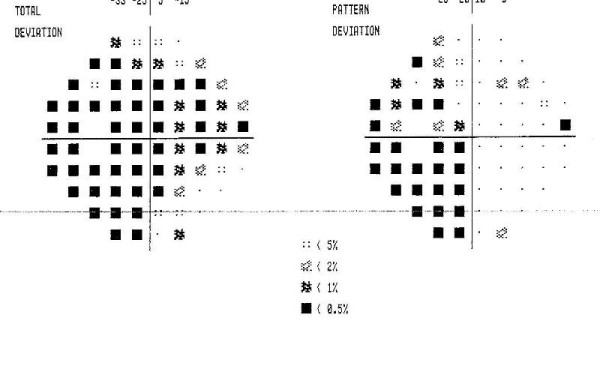
**Visual fields revealing left homonymous hemianopsia**.

Visual and auditory evoqued potentials did not show any delayed responses. Visual fields examination showed left homonymous hemianopsia (Figure 2).

**Figure 3:**
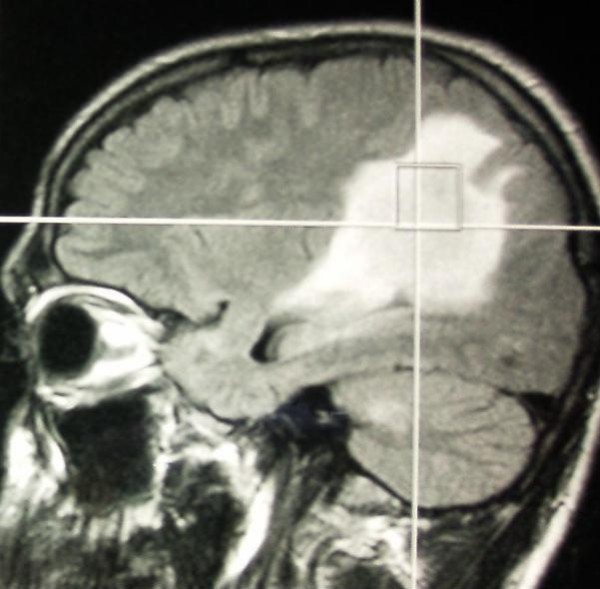
**MRI spectroscopy**.

**Figure 4:**
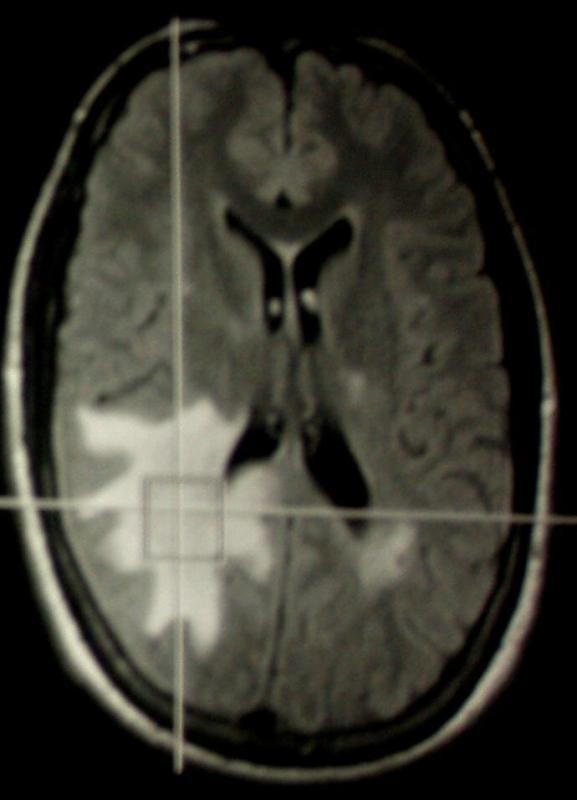
**MRI spectroscopy**.

**Figure 5:**
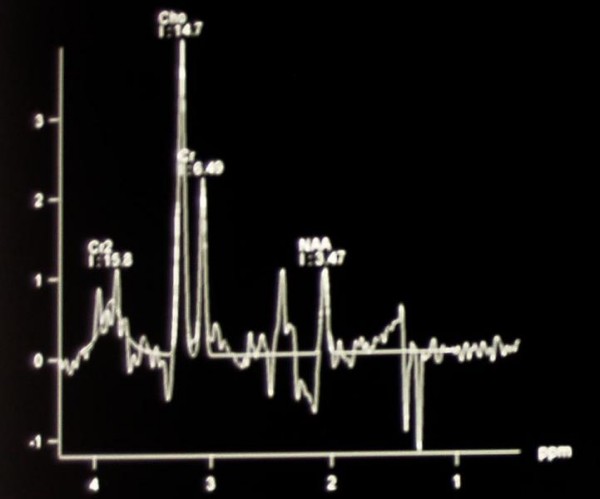
**MRI spectroscopy**.

After thorough examination of all clinical radiological and laboratory findings, (negative staging, increased signal intensity in T2W and Flair sequences, CSF examination), our differential diagnosis was mainly limited to a primary brain tumor, or to tumefactive Multiple Sclerosis. Accordingly, we proceeded to a Spectroscopic MRI [7]. The extensive lesion of the right hemisphere showed a decrease in the concentration of N-acetylaspartate and creatine, while a 50% increase in the concentration of choline was detected. No differences were found in the concentration of myoinositol between the two hemispheres. Several small lesions were found in the white matter of both cerebral hemispheres and the cerebellum, indicative of a demyelinating disease (Figure 3, Figure 4, Figure 5).

The patient received cortisol i.v., 1 gr Solumedrole i.v. for 5 days, substituted by per os administered cortisol. His neurological status gradually improved. During a five year follow up, he had no relapses and brain MRI showed a considerable decrease of the size of the tumor like demyelinating lesion.

## Discussion

Homonymous hemianopsia (HH) is the commonest form of acquired homonymous visual field defect. The most common causes of HH are stroke, head injury and intracranial tumors [8]. HH has been related to neurosarcoidosis [9]. Symptomatic homonymous visual field defects are relatively rare in MS, encountered in 1% of patients. On the other hand, single, rare, tumor-like MS lesions may present with uncommon symptoms, including HH [10-14].

In our case, the patient presented with left homonymous hemianopsia due to a demyelinating lesion of the right hemisphere mimicking brain tumour. Although he had no history of multiple sclerosis and hemianopsia is a relatively unusual first manifestation of this disease, oligoclinal bands were positive and IgG index was increased.

**Figure 6:**
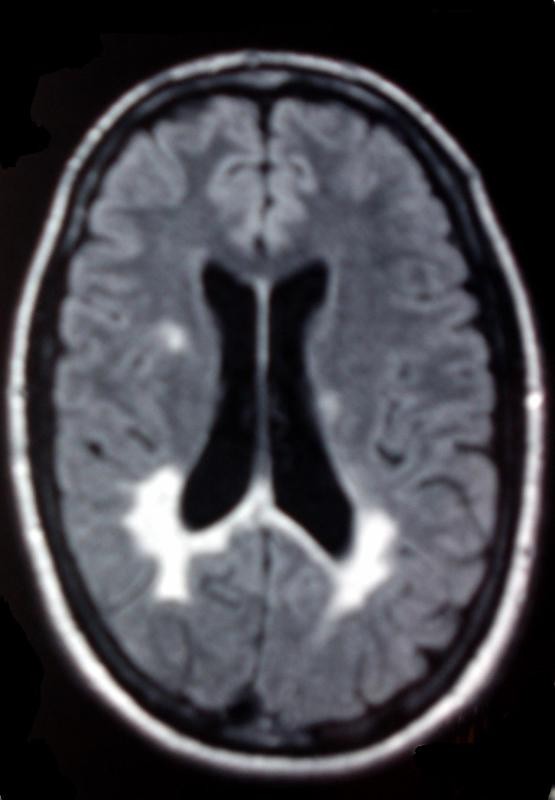
**MRI at 5 year follow-up**.

Visual fields were normal four months after the episode. During a five year follow up after the episode, the patient remained free of any neurological symptoms (Figure 6).

Diagnostically, MRI imaging of tumor like demyelinating lesions (TLDL) resemble brain tumors, and diagnosis is quite difficult [1]. The use of additional techniques may assist to distinguish the nature of these lesions. Perfusion-Weighted MRI (PWI), proton Magnetic Resonance Spectroscopy (MRS) as well as Thallium-201 Single-Photon Emission Computed Tomography (SPECT) and 18-FDG Positron Emission Tomography (PET), may be applied for an accurate differential diagnosis of a brain tumor [7]. In our case, MRS gave a detailed description of the lesion and assisted the final diagnosis. A common finding of the acute MS lesion is the reduced NAA levels due to axonal damage and neuronal mitochondrial dysfunction which is proposed to be reversible over time. Furthermore, elevation of choline is consistently found in acute MS lesions probably due to reactive astrogliosis and demyelination [15].

## Conclusion

Treating physicians should always bear in mind of tumefactive MS when encountering a cerebral lesion, since despite the demanding differential diagnosis by means of special techniques, this disease has a benign character and can be managed without exposing patients to the unnecessary risks of a surgical procedure.

## Abbreviations

CMV: cytomegalovirus; CSF: cerebrospinal fluid; CT: computer tomography; ECG: electrocardiogram; FDG: 18-fluorodeoxyglucose; HBV: hepatitis B virus; HCV: hepatitis C virus; HH: homonymous hemianopsia; HIV: human immunodeficiency virus; HSV1: herpes virus 1; HSV2: herpes virus 2; IV: intravenous; MRI: magnetic resonance imaging; MRS: proton magnetic resonance spectroscopy; PWI: perfusion-weighted MRI; SPECT: single-photon emission computed tomography; PET: positron emission tomography; VZV: varicella zoster virus.

## Competing interests

The authors declare that they have no competing interests.

## Authors' contributions

MEE, CP and CS were the treating physicians. MEE and DSE involved in acquisition of data. MEE, DSE, and CS involved in drafting of the manuscript. All authors read and approved the final manuscript.

## Consent

Written informed consent was obtained from the patient for publication of this case report and any accompanying images. A copy of the written consent is available for review by the Editor-in-Chief of this journal.
